# A New Type of Si-Based MOSFET for Radiation Reinforcement

**DOI:** 10.3390/mi15020229

**Published:** 2024-01-31

**Authors:** Weifeng Liu, Zhirou Zhou, Dong Zhang, Jianjun Song

**Affiliations:** 1 State Key Discipline Laboratory of Wide Band-Gap Semiconductor Devices and Integrated Technology, School of Microelectronics, Xidian University, Xi’an 710071, China; wfliu@mail.xidian.edu.cn (W.L.); 17792050036@139.com (D.Z.); jianjun_79_81@xidian.edu.cn (J.S.); 2 Radiation Resistant Integrated Circuit Technology Laboratory of China Aerospace Science and Technology Group Corporation, Xi’an 710071, China

**Keywords:** single-event effect, radiation-hardened, heavy ion, MOS devices

## Abstract

This paper thoroughly analyses the role of drift in the sensitive region in the single-event effect (SEE), with the aim of enhancing the single-particle radiation resistance of N-type metal-oxide semiconductor field-effect transistors (MOSFETs). It proposes a design for a Si-based device structure that extends the lightly doped source–drain region of the N-channel metal-oxide semiconductor (NMOS), thereby moderating the electric field of the sensitive region. This design leads to a 15.69% decrease in the charge collected at the leaky end of the device under the standard irradiation conditions. On this basis, a device structure is further proposed to form a composite metal-oxide semiconductor (MOS) by connecting a pn junction at the lightly doped source–drain end. By adding two charge paths, the leakage collection charge is further reduced by 13.85% under standard irradiation conditions. Moreover, the deterioration of the drive current in the purely growing lightly doped source–drain region can be further improved. Simulations of single-event effects under different irradiation conditions show that the device has good resistance to single-event irradiation, and the composite MOS structure smoothly converges to a 14.65% reduction in drain collection charge between 0.2 pC/μm and 1 pC/μm Linear Energy Transfer (LET) values. The incidence position at the source-to-channel interface collects the highest charge reduction rate of 28.23%. The collecting charge reduction rate is maximum, at 17.12%, when the incidence is at a 45-degree angle towards the source.

## 1. Introduction

The presence of high-energy particle radiation in space greatly affects the reliability of circuits and devices, particularly when it comes to single-event effects. The soft errors and hard errors caused by SEE have always been the focus of the industry [1,2,3]. The single-event effect occurs when the energy carried by high-energy particles is absorbed by the semiconductor material, causing the generation of numerous electron–hole pairs. These pairs disrupt the electrical characteristics of the devices themselves, leading to transient currents that flow into the post-stage circuits and causing various errors to occur [4]. As Moore’s law progresses and technology nodes shrink to 28 nm and below, the size of the channels in devices decreases due to factors like the short channel effect and proportional reduction. This causes the devices to exhibit more complex characteristics in terms of single-event effects [5]. At the same time, as the process node advances, the density of devices increases. This increase in density brings about problems such as the charge sharing effect, the micro-dose rate effect, and the multi-bit flipping effect [6,7,8,9,10]. As a result, both local and international researchers have concentrated on the problem of irradiation reliability in advanced nodes.

However, from the current domestic and international literature, the relevant research primarily focuses on hardening techniques and circuit-level SEE effects, such as SEE-resistant design and optimisation for Static Random-Access Memory (SRAM) and inverter chains [11,12,13,14,15,16]. On the other hand, device-level papers tend to analyse the phenomenon and mechanism of single-event effects in various devices, with very limited research on enhancing the anti-SEE capability of devices through structural improvements. Relevant studies have identified two main approaches to enhance the resistance of advanced node device-level anti-SEE settings. The first approach is substrate isolation, specifically through the use of a Silicon-On-Insulator (SOI) structure. This structure reduces the generation of electron–hole pairs caused by heavy-ion bombardment channels by isolating the substrate [12,17,18,19,20,21]. The second approach aims to improve the gate control capability by utilising a multi-gate Finned Field Effect Transistor (FinFETs), which has a smaller sensitive area. This reduces the probability of charge collection [20,22,23,24]. Additionally, structures such as the derived Gate-All-Around (GAA) [21,25] also exhibit excellent radiation resistance. However, these two ideas not only call for very expensive production and cutting-edge technology but they also create new issues: the SOI structure requires additional insulation layer processing, and, due to the floating body effect, the bipolar amplification effect is much stronger than that of planar MOS devices [2]. On the other hand, the complex gate shape of FinFET devices gives rise to increasingly intricate irradiation issues, such as the micro-dose rate impact [7].

This study presents a design scheme for determining the length of the Lightly Doped Drain (LDD) zone. The technique is based on moderating the electric field in the sensitive region by thoroughly examining the role of charge collection in the sensitive region of the single-event effect NMOS. The proposed scheme is different from the ideas described above. Although this approach decreases the amount of charge collected at the drain end of the device, it also leads to a degradation in the driving current. This research proposes the use of a 28 nm LDD_pn device to resolve the problem. The device is a composite MOS structure with lightly doped source–drain end-tethered pn junctions, designed based on the principle of charge collection shunt. The paper also includes a simulation study of the device’s electrical characteristics and single-particle transient current. The study looks into the aspects that determine the amount of improvement in a novel structure’s anti-SEE performance under varied heavy-ion incidence conditions and device characteristics. It determines the range of detailed device parameters that result in optimal anti-SEE capability, presenting a novel design concept for advanced node anti-SEE devices.

## 2. Device Design Principle and Simulation Result Analysis

### 2.1. Principle of Device Structure Design

Various studies have shown [2,4,5] that the single-event effect in MOS devices involves three separate stages of charge collection: 1. The transient drift process, where the strong electric field formed by the reverse-biased pn junction at the sensitive region (the interface between the drain and the channel) rapidly extracts the charge generated by the heavy-ion incidence, and the transient drift charge component is $Q_{D}$; 2. Transient accumulation process; this process basically involves many charges interfering with the space charge region of the pn junction’s during the drift phase, which extends and enhances the electric field. As a result, more charges are accumulated, increasing the total number of collected charges and creating a transient accumulated charge component of $Q_{F}$; 3. Delayed diffusion process; a part of the charge ($Q_{DF}$) generated at a farther position takes longer to be collected, which is manifested as the tail of the collection current. This is because of the diffusion effect of charge concentration differences, which takes some time. By integrating the single-event transient current, the following equation is provided:

(1) $$Q_{Collected}=\int_{0}^{T}I_{d}dt=Q_{D}+Q_{F}+Q_{DF}$$

The integral result is the collected charge at the drain end, and the upper limit of integral *T* is 10^−8^ s, at which time the transient current approaches zero.

The above analysis points out that the electric field near the sensitive area is the main parameter that affects the drift effect. As indicated by the above analysis, mitigating the electric field in the sensitive area can enhance the device’s anti-SEE capabilities. According to the following equation:

(2) $$E_{x}=\frac{U}{d_{x}}$$

In the formula, $E_{x}$ is the electric field strength and *U* is the potential difference between two points in a uniform electric field. The length of the RELATIVELY LDD region, i.e., $d_{x}$, can be adjusted to mitigate the transverse electric field idensity. Based on this electric field mitigation theory, this paper proposes a RELATIVELY LDD area growth device; however, according to the general equation for the channel carrier velocity motion versus electric field and mobility [26]:

(3) $$V\left(x\right)=E_{x}*\mu$$

The decrease in the electric field causes the channel carrier velocity to decrease, which affects the channel current and makes the driving current of the RELATIVELY LDD area growth device decrease. Consequently, more advancements in the RELATIVELY LDD area growth device are required.

It is also noted that, for the single-event transient collection charge of the MOS device, the shunt direction is the source, drain, and substrate directions. The number and types of shunt charges are different because the potentials of these three ports are different. Based on this idea, adding additional port shunts above the source and drain RELATIVELY LDD regions minimises the charge collected at the sensitive regions. In order to avoid large leakage currents generated by the additional port affecting the electrical characteristics of the device, a reverse-biased pn junction can be introduced as an additional shunt port. This reverse-biased pn junction is close to the RELATIVELY LDD region, so it can also change the electric field in the RELATIVELY LDD region. This helps to mitigate the current drop caused by the excessively long length of the RELATIVELY LDD region, which in turn reduces both the peak single-event transient current and the tail collection current.

### 2.2. Device Simulation Model

**Figure 1:**
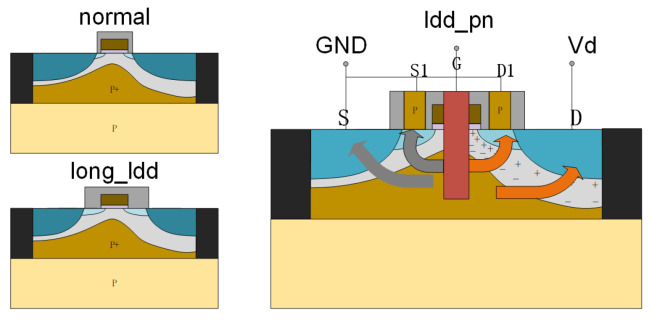
(Network color map) reference NMOS device structure (normal and long_relatively LDD) and relatively LDD_pn device structure schematic diagram.

According to the 2.1 device design principle, this paper adopts the Sentaurus TCAD (Technology Computer Aided Design) series software of Synopsys to design the structure of the device, and the 28 nm NMOS device diagram is shown in Figure 1.

In this paper, a novel improvement has been made to the relatively LDD_pn device, which surpasses the conventional MOS device. The enhancement involves the introduction of additional pn structures as electrodes for shunting, i.e., the Source1 (S1) and Drain1 (D1) poles shown in Figure 1. The figure also illustrates four arrows that represent the four charge shunting paths. The device is on a silicon substrate with a 2 nm silicon oxide dielectric and a 2.2 nm hafnium oxide dielectric for the gate dielectric layer. Channel doping and dielectric thickness can adjust the device threshold voltage. The channel size is 28 nm, and the source–drain region adopts N-type heavy doping. The Si_3_N_4_ sidewall is deposited and etched on both sides of the gate. Subsequently, P-type silicon is precipitated on both sides to generate the S1 and D1 poles. Finally, the second layer of sidewall is deposited and etched. In order to reduce the effect of source–drain heavy doping on the channel, adjust the interface position of the pn junction while corresponding to the two sidewalls, and increase the driving current of the device; a double lightly doping source–drain process is adopted, with the first doping concentration of 1 × 10^19^ cm^−3^ and the second doping concentration of 5 × 10^19^ cm^−3^. Specific simulation parameters are shown in Table 1.

**Figure 2:**
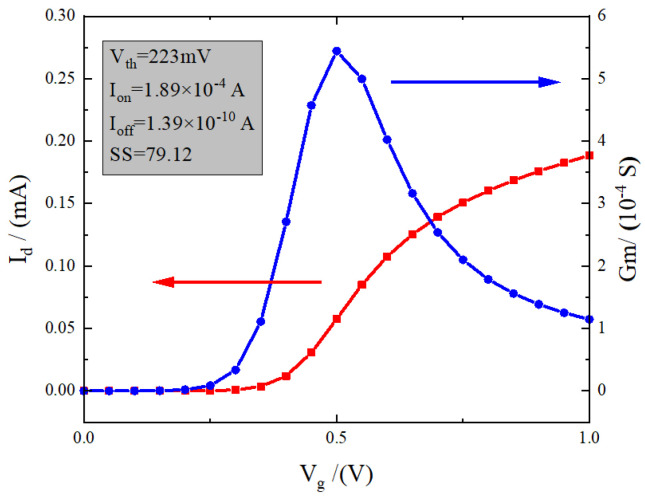
(Network version color map) relatively LDD_pn device transfer characteristic curve and transconductance curve.

The TCAD tool is utilised to simulate the electrical properties of the device. The device’s D-terminal voltage, V_*d*_, is set to 1 V as the default value. The gate voltage, V_*g*_, is set to 0 V. Additionally, the electrodes D1 and S1 are grounded to 0 V. The corresponding transfer characteristic curve (V_*d*_ = 0.1 V) and transconductance curve are shown in Figure 2. The gate work function is set to 4.2 eV, which was adjusted to provide a device threshold voltage of 226 mV. The device has a good driving circuit with an on-state current of 0.189 mA, an off-state current of 1.39 × 10^−10^ A, and a peak transconductance of 5.4 × 10^−4^ s. The device has a good subthreshold characteristic, with a subthreshold swing of 79.12. The good subthreshold characteristic can reduce the influence of the leakage current on the collected charge at off-state. This enables the examination of the most severe irradiation damage caused by the heavy-ion incidence.

**Figure 3:**
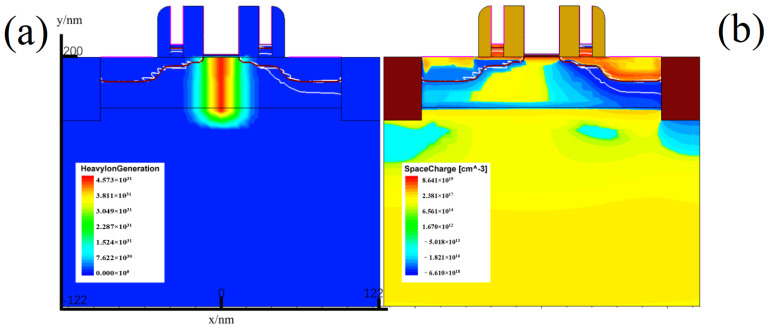
(Network color map) (**a**) heavy-ion production rate at the time of heavy-ion incident; (**b**) space charge distribution at the time of heavy-ion incident.

The heavy-ion incidence model provided by the TCAD tool is a commonly used simulation model to study the single-event effect. The default parameters of this model and some important physical models are shown in Table 2. The spatial information of heavy-ion incidence can be simulated by parameters such as position, direction, etc.; the carrying energy of heavy-ion incidence can be simulated by LET, length, and width; and the time parameter can be used to control the time required for the heavy ion to penetrate into the material [27]. The time parameter controls the time required for heavy ions to penetrate into the material. It is worth noting that the model uses the LET value as the default parameter, and the conversion relationship between the two units of this parameter is [28]

(4) $$1\;\mathrm{pC}/\mu m=\frac{1\times 10^{-12}\;C}{1\times 10^{-12}\;C/\mathrm{pair}}\times \frac{3.6\;\mathrm{eV}/\mathrm{pair}}{\rho \times 10^{6}}\times 10^{4}=96.608\;{\mathrm{MeV}\cdot \mathrm{cm}}^{2}/\mathrm{mg}$$

where the density of the semiconductor material is characterised by ρ in the formula, and the density of silicon is 2.33 g/cm^3^. Using the structural parameters of Table 1 for simulation, the heavy-ion generation rate and space charge region distribution image at the moment of heavy-ion incidence is shown in Figure 3, which clearly shows that the heavy ions are incident from the channel, with an incident depth of 50 nm, which produces a cylindrical ionisation region, and the electron–hole pairs generated from this disrupt the space charge distribution, resulting in the formation of a “funnel”-shaped distribution of space charge at the channel in Figure 3. At the same time, due to the presence of a high potential in the sensitive region (drain), this rapidly attracts a large number of electrons, resulting in an asymmetric deformation of the space charge “funnel”.

### 2.3. Simulation Results Analysis of RELATIVELY LDD Region Growing Device Based on Electric Field Relaxation Theory

**Figure 4:**
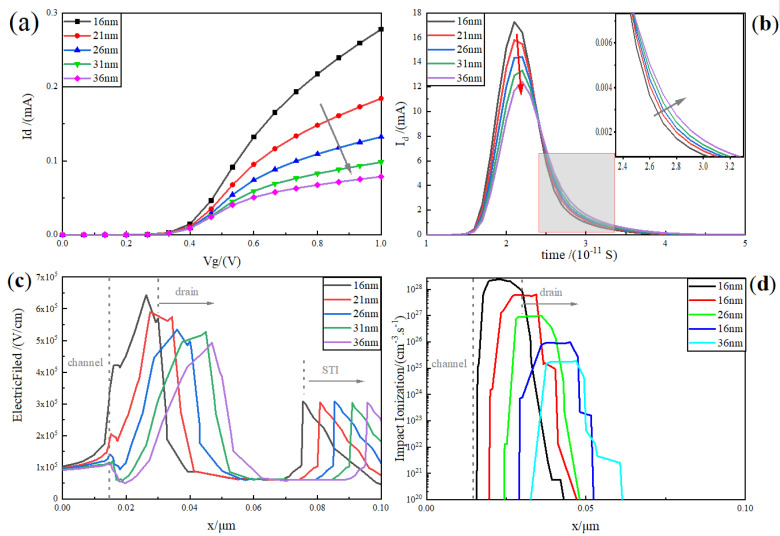
(Colour online version) (**a**) plot of the variation in the transfer characteristics of the device (Vd = 0.1 V) with increasing RELATIVELY LDD; (**b**) single-particle transient current profile of the device for heavy-ion incidence at a LET value of 1 pC/μm; (**c**) one-dimensional transverse electric field distribution (y = 1 nm) (from channel to STI region); (**d**) plot of one-dimensional collisional ionisation rate for heavy-ion incidence (y = 1 nm).

The RELATIVELY LDD process is an indispensable process for deep submicron planar MOS, which is helpful to improve the leakage current of the device, threshold voltage instability, hot carrier effect, and a series of short channel effects [29]. Figure 4a shows the device transfer characteristics with the increase in the length of the RELATIVELY LDD region. It is clearly visible that, with the increase in the RELATIVELY LDD region, the driving current of the device gradually slows down, and the current at 1 V decreases from 0.28 mA to 0.079 mA. The increase in the length of the RELATIVELY LDD region specifically results in a reduction in the transverse electric field and transverse electric potential intensity while increasing the resistance between the channel and the drain. Consequently, this leads to a decrease in the driving current.

Unlike the driving current trend, Figure 4b shows the single-event transient current curve of the heavy-ion incident device with a LET value of 1 pC/μm. It is easy to see that, as the length of the RELATIVELY LDD region increases, the peak value of the transient current decreases gradually. The peak transient current decreases from 17.27 mA at 16 nm to 12.41 mA at 36 nm, which implies that the charge collected by the drain drift is gradually decreasing. The slight increase in the length of the RELATIVELY LDD also means that the path required for diffusion from the centre of the channel increases, which is manifested by a slight increase in the tail current of the pulse curve with the increase in the RELATIVELY LDD length.

Further, from the analysis of the one-dimensional transverse electric field distribution plot of the device in Figure 4c, it can be seen that the electric field curve of the sensitive region (channel to drain region) gradually slows down with the increase in the RELATIVELY LDD, while the peak value also decreases gradually. In addition, Figure 4d demonstrates the one-dimensional impact ionisation rate diagram during heavy-ion incidence, and it is clearly visible that the peak impact ionisation rate peak decreases from the order of 10^28^ cm^−3^·s^−1^ to the order of 10^25^ cm^−3^·s^−1^, which exhibits the same decreasing trend as that of the peak electric field. This indicates that increasing the length of the RELATIVELY LDD region can effectively moderate the electric field and impact the ionisation rate in the sensitive region, thus improving the device’s resistance to the single-event effect.

The above analysis indicates that increasing the RELATIVELY LDD region length is a double-edged sword, so additional processing is required to balance the driving current density and the ability to resist single-event transients.

### 2.4. Simulation Results Analysis of Relatively LDD_pn Improved Device Based on Shunt Theory

**Figure 5:**
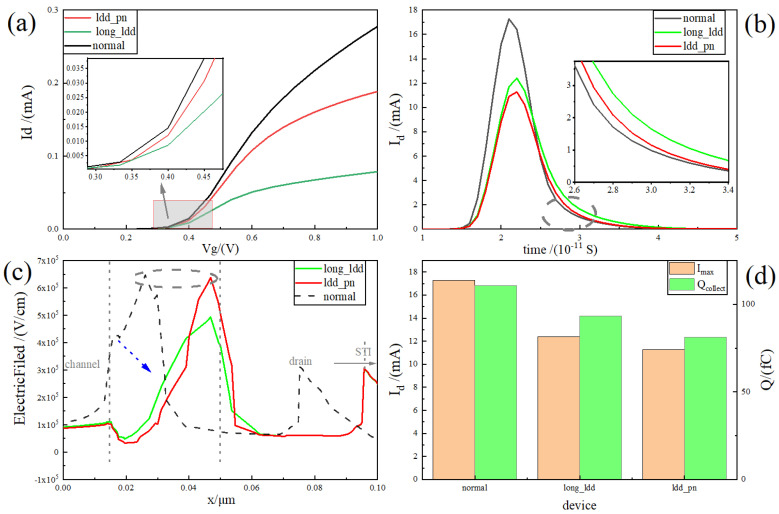
(Network version color map) (**a**) transfer characteristic current comparison diagram; (**b**) comparison diagram of transverse electric field distribution; (**c**) comparison diagram of transient collecting current (from channel to STI region); (**d**) comparison of transient current peak and total collected charge.

In order to balance the driving current strength and the ability to resist the single-event effect, this paper introduces the relatively LDD_pn structure shown in Figure 1, for which the electrical and irradiation characteristics are simulated in this section. Figure 5 comprehensively shows the comparison of transfer characteristics (Figure 5a, one-dimensional transverse electric field distribution (Figure 5b, transient collection current (Figure 5c, and peak transient current and total collection charge (Figure 5d, respectively, with the normal device and the long_relatively LDD device as the reference case.

Figure 5a shows that the relatively LDD_pn junction structure can effectively reduce the forward current attenuation brought about by the excessively long_relatively LDD region length. This makes the device current curve better than the long_relatively LDD device. It can be clearly seen that the current of the relatively LDD_pn device can reach 0.18 mA at 1 V, although not as good as the normal device of 0.26 mA, but much higher than the long_relatively LDD device of 0.079 mA, effectively alleviating the phenomenon that the driving current decreases with the increase in RELATIVELY LDD region length.

The transient collection current plot shown in Figure 5b indicates that the relatively LDD_pn device has the lowest current peak. The tail current curve is also very close to that of the normal device, which suggests that the tail diffusion current is much better when the RELATIVELY LDD is too long. Figure 5c shows the distribution of the one-dimensional transverse electric field for each case. It is clearly visible that the electric field peak of the relatively LDD_pn structure is close to that of the normal device, while the electric field at the junction of the channel and RELATIVELY LDD is slightly lower than that of the long_relatively LDD device. The moderate channel electric field can reduce the drift velocity of charge and improve the recombination rate at the same time. The electric field at the junction can collect the tail diffusion current quickly, which makes the RELATIVELY LDD_pn structure both improve the current and further enhance the ability to resist the single event of the device.

Figure 5d shows in more detail the variation in peak transient current and collected charge with different devices. It can be clearly seen that the peak transient current of the device with the relatively LDD_pn structure is 11.27 mA, and the total collected charge is 81.12 fC. Compared with the normal device, the current peak is reduced by 34.7%, and the collected charge is reduced by 25.23%; compared with the long_relatively LDD device, the peak current is reduced by 7.17%, and the total collected charge is reduced by 13.85%. This structure can enhance the device’s single-event resistance characteristics as well as the current attenuation brought on by RELATIVELY LDD growth.

**Figure 6:**
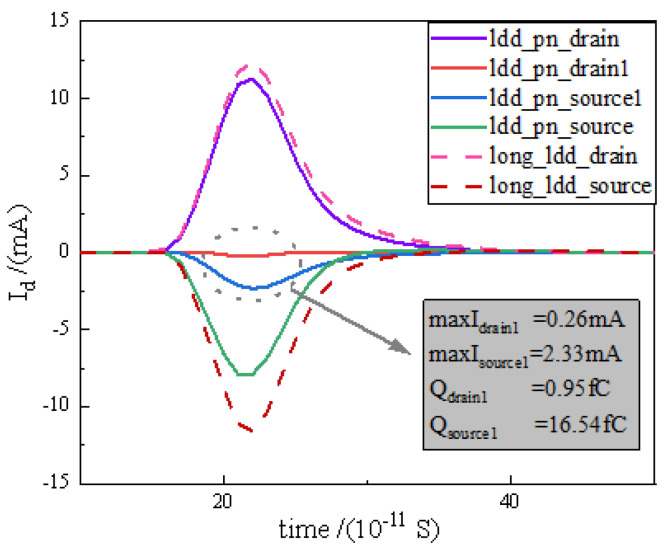
(Colour figure for web version) transient current profiles for Drain, Source, Drain1, and Source1 electrodes of relatively LDD_pn structured device and Drain and Source electrodes of long_relatively LDD device.

Figure 6 shows the transient current profiles of the D, S, D1, and S1 electrodes of the relatively LDD_pn structured device. These profiles are compared to the source and drain transient current curves of the long_ relatively LDD device. The lower transient current profiles of D1 and S1 are clearly visible. This means that the additional introduced electrodes have a shunting effect, which can significantly reduce the number of collected charges at the source and drain. The peak transient current of the D1 pole is 0.26 mA, and the collected charge is 0.95 fC, while the peak transient current of the S1 pole is 2.33 mA, and the collected charge is 16.54 fC, which indicates that, due to the influence of the potential, the S1 has a stronger shunt capacity than the D1.

In summary, the improved structure can effectively improve the current characteristics in the case of excessively long RELATIVELY LDD regions and further enhance the ability of the single-event effect of the device.

## 3. Improvement in Device Single-Event Effect Influence Factor Analysis

This section focuses on the single-event effect of the improved device with relatively LDD_pn structure under different LET values, different incident positions, different incident depths, different P-zone gasket parameters, different potential settings, and other factors. The structure is also compared with a normal device and a long_relatively LDD device to discuss the influencing factors of the improvement in anti-SEE ability, and the detailed device parameter range under the best anti-SEE ability is determined.

### 3.1. Influence of Different LET Values on the Single-Event Effect of the Device

**Figure 7:**
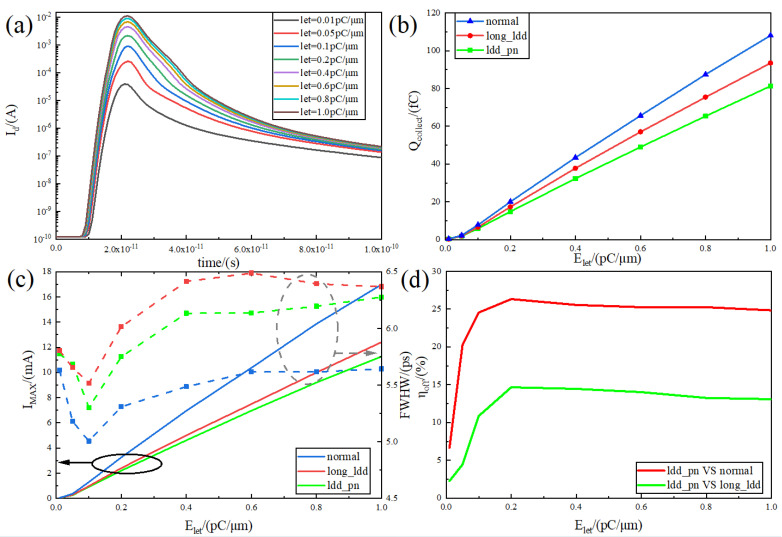
(**a**) Transient current versus let value; (**b**) variation in collected charge versus let value for relatively LDD_pn, normal, and long_relatively LDD devices; (**c**) variation in peak current and pulse width (FWHW) versus let value for relatively LDD_pn, normal, and long_relatively LDD devices; (**d**) variation in collected charge reduction rate versus let value for relatively LDD_pn, normal, and long_relatively LDD devices. Variation in collection charge reduction rate with let value for relatively LDD_pn device, normal device, and long_relatively LDD device.

The number of carriers generated by single-event incidence is related to the energy carried by the particle, its mass, the density of the semiconductor material, etc., and reference [27] pointed out that the formula for the number of charges generated by single-particle incidence is

(5) $$\frac{dN}{dx}=\frac{dP}{dx}=\frac{\rho }{3.6\;\mathrm{eV}/\mathrm{pair}}\times E_{let}$$

where *N*, *P* are the number of electrons and holes, respectively, ρ is the density of the semiconductor material, the density of silicon is 2.33 g/cm^3^, and 3.6 eV is the average ionisation energy generated by electron–hole pairs in silicon. It can be seen that the number of generated charges is linearly correlated with the linear energy value Elet. The simulation uses the default settings of Section 3 to study the relationship between the collected charge at the drain and pulse current and the linear energy value under the turn-off condition. Considering that the linear energy value in space [30] can be up to more than 90 MeV·cm^2^·mg^−1^, the upper limit of scanning is set to 1 pC/μm (converted from Equation (1) to approximately 96 MeV·cm^2^·mg^−1^), and the initial value is 0.01 pC/μm, with the incidence position at the centre of the channel. The image of the transient current variation with Elet obtained from the simulation is shown in Figure 7a.

From Figure 7a, the collection current curve moves up as the linear energy value goes up. The peak value is gradually increased from the lowest point of 40 μA to 11.3 mA. The peak position is also slightly shifted backward from 21 ps to 23 ps, which is attributed to the fact that the increase in the linear energy value increases the particle range. The electron–hole pair peak concentration position in the silicon material is also shifted downward, and the particles need more time to move to the drain pole to be collected.

Figure 7b compares the variation in the collected charge with the linear energy value for the relatively LDD_pn device with the two reference devices in more detail. It can be seen that there is a linear relationship between the collected charge and the linear energy value, which is consistent with the analysis in Equation (5). For the normal device and long_relatively LDD device, the difference in the recombination rate of the pn junction depletion region is caused by the difference in the electric field and potential of the devices, and the diffusion also produces more recombination due to the longer diffusion movement distance of the long_relatively LDD device. This causes long_relatively LDD to have an overall lower slope charge collection curve. The relatively LDD_pn device introduces a new charge path as in Figure 6 while improving the RELATIVELY LDD electric field, so the overall collected charge curve further decreases. The relationship between peak current and pulse width (FWHW) between the relatively LDD_pn device and the two reference devices is further analysed in Figure 7c.

As seen in Figure 7c, the peak currents of the three devices show an approximately linear relationship. For the same device, the peak collection current at the drain can be expressed as [31]

(6) $$I_{peak}=\frac{E_{let}}{3.6}\frac{q^{2}\mu_{n}N_{A}}{\varepsilon }\times X_{p}=E_{let}k$$

where $\mu_{n}$ is the dielectric constant, $N_{A}$ is the drain doping concentration, ε is the dielectric constant of Si, and $X_{p}$ is the length of the space charge region of the pn junction; for the same device under the same conditions, these parameters can be approximated as constants, and thus the peak current has an approximately linear relationship with the LET value. In addition, it can be seen that the peak relatively LDD_pn transient current curve in Figure 7c is much improved compared to the normal device, which is precisely caused by the introduction of the subgate pn junction to improve the electric field and potential at the interface between RELATIVELY LDD and the channel, as well as the introduction of the additional charge path. It can also be noticed that the normal device has a lower FWHW overall compared to the other devices; this means that the normal device has a lower tail current, which is consistent with the trend in Figure 5c. The lengthened RELATIVELY LDD region makes the charge take longer diffusion time, while the reduced electric field makes the charge movement rate decrease, resulting in slower charge diffusion speed and longer collection time, so the tail current caused by diffusion increases, resulting in an increase in FWHW. The relatively LDD_pn device has a higher peak electric field, which makes its tail collect the charge faster compared to the long_relatively LDD device, and it therefore has a lower FWHW.

Figure 7d compares the variation in the collection charge reduction rate of the relatively LDD_pn device with that of the normal device and long_relatively LDD device. The collection charge reduction rate efficiency equation is

(7) $$\eta =\frac{|Q_{a}-Q_{b}|}{Q_{a}}\times 100\%$$

where $Q_{a}$, $Q_{b}$ are the number of charges collected by different devices. Figure 7d shows that the two curves show the same trend, the efficiency curve as a whole increases rapidly with the increase in LET value, and there is a maximum value at 0.2 pC/μm, and then the curve decreases slowly and tends to level off. The peak collected charge reduction rate of the relatively LDD_pn device at 0.2 pC/μm is 26.33% compared to the normal device and 14.65% compared to the long_relatively LDD device, which shows that the structure proposed in this paper has a good anti-radiation ability compared to normal and long_relatively LDD devices. Meanwhile, the efficiency tends to be stable between 0.2 pC/μm and 1 pC/μm, which is the common range of LET values for ground-to-space radiation [30], indicating that the device in this paper has a wide range of anti-radiation applications.

### 3.2. Influence of Different Incidence Positions on the Single-Event Effect of the Device

**Figure 8:**
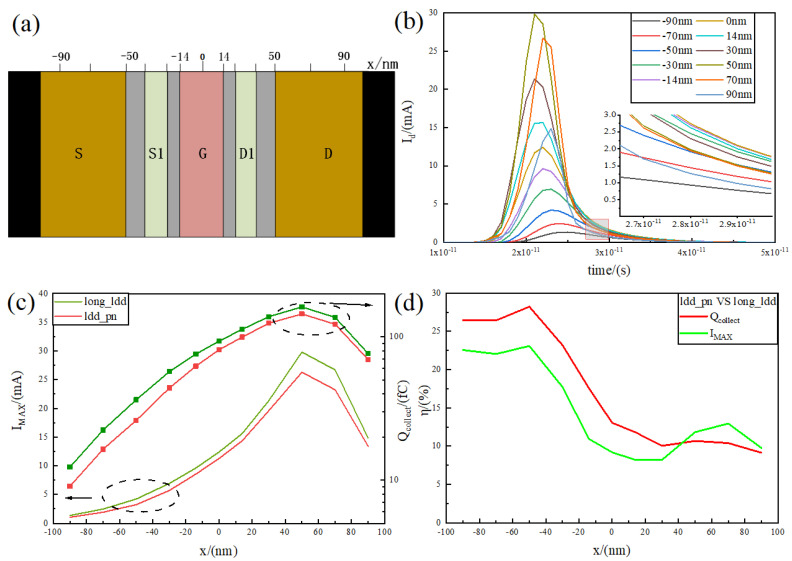
(**a**) Heavy-ion incident position distribution, from −90 nm to 90 nm; (**b**) transient current images of relatively LDD_ pn device at different incident positions; (**c**) transient current peaks and charge collection images at different incident positions of relatively LDD_ pn and long_relatively LDD devices; (**d**) the transient current peaks at different incident positions of the relatively LDD_ pn device and the long_relatively LDD device and the collection of electrical reduction rate images.

Due to the different electric fields and potentials at different positions of the device, it results in different sensitivities for collecting charge at different positions. In this paper, 11 points were chosen at random from −90 nm to 90 nm as shown in Figure 8a, which include the source and drain, RELATIVELY LDD, the channel centre, and other important spots. The paper then looks at how sensitive these points are to the collected charge, and the results of the collected current are shown in Figure 8b. The maximum value of the peak current appears at 50 nm, where the current peak is 26.3 mA, which is exactly the junction of the RELATIVELY LDD region and the drain and is the most sensitive area of the device to the single-event effect. At the same time, due to the tail current being mainly affected by the diffusion motion, the charge far away from the sensitive area is continuously recombined during the diffusion process, and the time required to collect the charge moving to the RELATIVELY LDD area and the drain junction area is different, which makes the tail current also change with the incident position.

The long_relatively LDD device with the same size is selected as a comparison to further analyse the improvement in peak current and collected charge, and the results are shown in Figure 8c. It can be seen that the peak current and collected charge of both are basically in the same trend with the transverse position, gradually increasing from −90 nm to 50 nm, with a peak at 50 nm and then decreasing to 90 nm. This again indicates that 50 nm is the most sensitive area of the device. At the peak, the collected charges of the relatively LDD_pn device and the long_relatively LDD device are 144 fC and 163 fC, respectively, and the peak currents are 26.3 mA and 29.9 mA, respectively, which shows that the relatively LDD_pn structure also has good radiation resistance in the sensitive area. The collection charge and peak current reduction efficiencies of the relatively LDD_pn compared to the long_ relatively LDD device at different locations are analysed, and the results are shown in Figure 8d.

Figure 8d shows that the collected charge and peak current reduction efficiencies are different at different positions, and the maximum value of both occurs at −50 nm, with the highest collected charge reduction efficiency of 28.23% and the highest peak current reduction efficiency of 23.10%. This is exactly the junction of the source and the RELATIVELY LDD, where the electric field is stronger compared to the surrounding area. The source and S1 poles can quickly extract the electron–hole pairs created here when coupled with the relatively LDD_pn structure, which further reduces the charge diffused to the drain and lowers the collected charge. Furthermore, the strong electric field at 50 nm makes it hard for D1 to collect the charge because the source-to-drain electric field is not uniform. This, along with the fact that many charge generation positions are in the sensitive area, leads to a lower efficiency at 50 nm than at −50 nm, which is only 10.67%. It is also noted that the charge collection efficiency curve is overall higher than the peak current efficiency curve before 50 nm, while the opposite is true after 50 nm. The drift effect is the main cause of the peak current. The drift effect is stronger when the sample is close to the sensitive region. On the other hand, the diffusion effect is stronger when the sample is farther away from the sensitive region. This means that there is less charge recombination, which increases the overall collected charge efficiency and makes the transient tail current smoother, which is in line with the trend shown in Figure 8b.

### 3.3. Influence of Different Incident Angles on the Single-Event Effect of the Device

**Figure 9:**
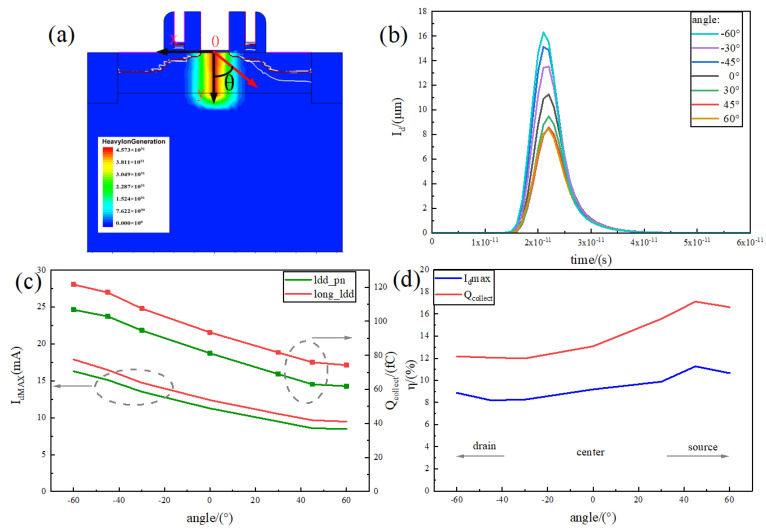
(**a**) Heavy-ion incident angle position coordinates, from −60 ° to 60 °; (**b**) transient current images of relatively LDD_ pn device at different incident angles; (**c**) the transient current peaks and charge collection images of relatively LDD_ pn device and long_relatively LDD device at different incident angles; (**d**) the transient current peaks of relatively LDD_ pn devices and long_relatively LDD devices at different incident angles and the collection of electrical reduction rate images.

The heavy-ion incident direction in space is variable, and there may exist multiple incident angles, yet TCAD supports the simulation of the heavy-ion injection at different incident angles. Considering the coordinate axis as in Figure 9a, with the y-axis as the initial incident angle of 0°, seven incident directions, such as −60° (pointing to the drain direction) to 60° (pointing to the source direction), are set. The relationship between the transient current and time is shown in Figure 9b. The figure shows that the transient current curve as a whole is gradually decreasing as the angle changes from −60° to 60°, and the peak transient current decreases from 16.31 mA to 8.47 mA. On the one hand, when the degree increases in the negative direction (towards the drain), as pointed out in Equation (8):

(8) $$V=\frac{16\pi }{cos\;\theta }$$

As the incident angle θ increases, the volume formed by the incident also increases, i.e., the greater the number of electron–hole pairs generated by collision ionisation. On the other hand, these electron–hole pairs are generated closer to the sensitive area, and the charge motion distance is shorter. Both factors cause the transient current to increase as the degree increases in the negative direction. In the positive and negative directions (towards the source), although the number of carriers generated also increases, the position of electron–hole pairs will also be farther away from the sensitive area and also affected by the shunt effect of the S-pole and the S1-pole, which is what makes the current decrease when it changes in the positive direction.

Further comparative analysis of the current peak and the curve of the collected charge with the angle of the device and the long_relatively LDD device is shown in Figure 9c. It can be seen that the current peak and the collected charge are decreasing as a whole, which is consistent with the trend of the Figure 9b curve. At the same time, the current peak and the collected charge of the relatively LDD_pn device are lower than the corresponding curves of the long_relatively LDD device. This indicates that the device still has good radiation resistance from different heavy-ion incident angles.

Considering the peak current and collected charge reduction rate as shown in Figure 9d, it can be seen that both curves follow the same trend, increasing slowly to a maximum at 45° and then decreasing. The peak current reduction rate at 45° is 11.27% and the collected charge reduction rate is 17.12%. This means that there is a higher reduction rate when the incident angle is biased towards the source. The overall collected charge reduction rate remains above 12% at all angles, which fully demonstrates the excellent anti-SEE performance of the device.

### 3.4. Influence of PN Junction P-Type Doping Region Parameters on Single-Event Effect

**Figure 10:**
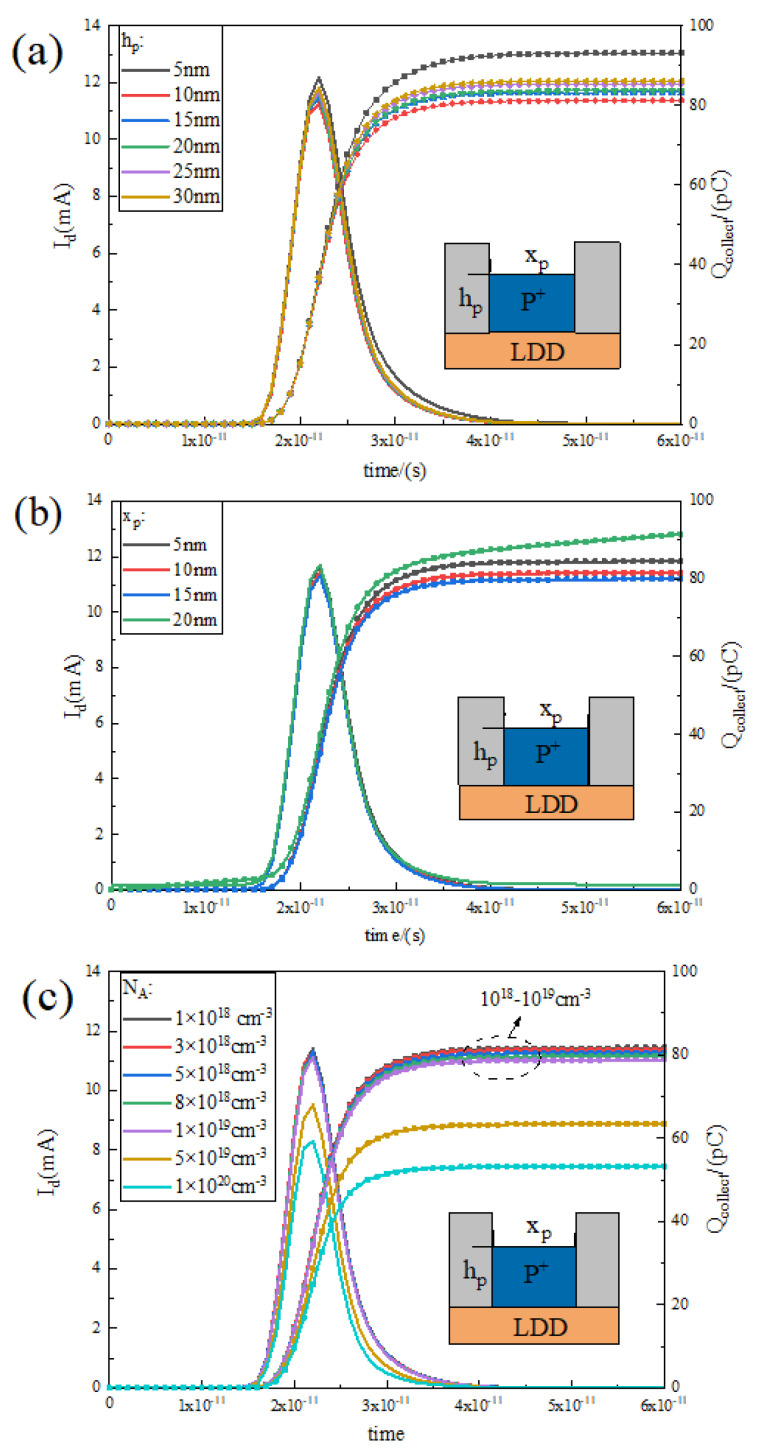
Transient current and collected charge distribution curves with time for different P-region heights h (**a**), lengths x (**b**), and doping concentrations (**c**).

Since the introduction of an additional P-type doping region above the RELATIVELY LDD region and on both sides of the gate changes of the structure of the device, it is necessary to discuss the influence of the size and doping parameters of the region on the SEE resistance of the device. The effect of P-type doping on the transient current is analysed in Figure 10a,b for the case of different thicknesses h and different widths x, respectively, and the curve of the collected charge curve with time is analysed. As can be seen from Figure 10a, the transient current curve and the collected charge curve at 5 nm high are higher than the other size cases, indicating that too-thin P-type doping is not favourable for the suppression of transient current. From 10 nm onwards, the collected charge and transient current are slowly increasing with increasing size, but still lower than the data at 5 nm. Obviously, 10 nm is a better size, but thicker P-type doping is acceptable considering the fabrication process.

Similarly, consider the variation in transient current and collected charge curves under variation in P-type doping width x. It is clearly seen in Figure 9b that the number of collected charges is decreasing and then increasing as the x size increases, with a minimum at 15 nm. Too narrow x is not favourable for electrode contact and process preparation, while too wide x will make the P-type doping area cover the source–drain region, which may produce a certain leakage current and is not conducive to the rapid reduction in the tail diffusion current.

Then, considering the transient current curves and collected charge curves under different doping concentrations as shown in Figure 10c, it is obvious that the transient current curves and collected charge curves are gradually shifted downward with the increase in doping concentration; the overall change in the two curves is not significant when the doping concentration is between 10^18^ cm^−3^ and 10^19^ cm^−3^, and, when the doping concentration continues to increase to 10^20^ cm^−3^, the two curves have an obvious downward shift, which means that the total number of charges collected at the drain is decreasing rapidly. However, when the doping concentration in the P-type region is too high, higher than the RELATIVELY LDD doping concentration, it will make the P-type doping erode the RELATIVELY LDD region and greatly affect the electrical characteristics of the device, which can be avoided by adding the intrinsic region between the RELATIVELY LDD and P-type regions to adjust the interface position of the pn junction.

### 3.5. Influence of Electrode and Potential Conditions on Single-Event Effect

**Figure 11:**
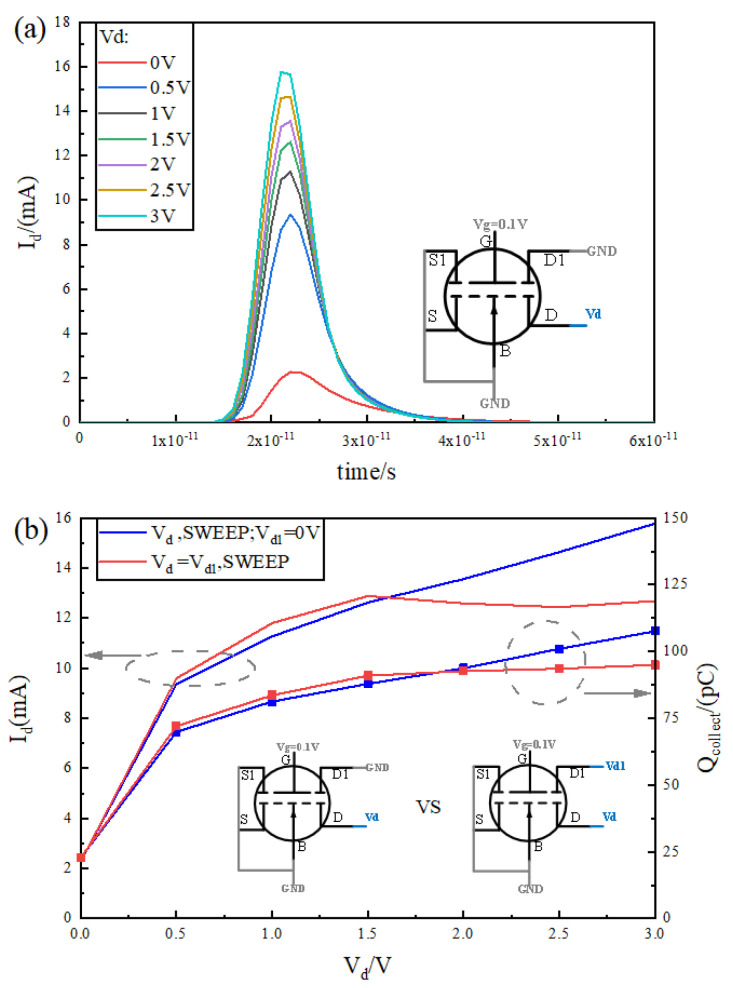
(**a**) Curve of transient current distribution with time at $V_{d1}$ = 0 V for different values of $V_{d}$; (**b**) curve of transient current and collected charge at $V_{d1}$ = $V_{d}$ and $V_{d1}$ = 0 V as a function of $V_{d}$.

It is important to think about how different potential cases for different electrodes affect the anti-SEE properties of the device in this paper because it has introduced additional Drain1 (D1) and Source1 (S1) electrodes. To avoid the effect of S1 bias on the device, the S1 and S poles and B are grounded; meanwhile, the gate voltage is set to 0.1 V, consistent with the previous section. Since D1 is close to D, there exist two settings for the voltage at D1. One is grounded, which puts the pn junction at D1 in a reverse-biased state; the other is to set D1 and D together to a uniform voltage, which puts the pn junction at D1 in a bias-free state. Figure 11a is the curve of the transient current with the change in Vd voltage when D1 is grounded. As Vd increases from 0 V to 3 V, the transient current curve gradually shifts upward, and the charge collected at the drain is gradually increasing. Note that, at 0 V, although there is no potential difference between source and drain, the existence of the charge path makes it possible to still have a certain transient current at this point.

Figure 11b demonstrates the relationship between the peak transient current and the number of collected charges with voltage for both $V_{d1}$ = 0 V and $V_{d1}$ = $V_{d}$ settings. Peak transient current and the total number of collected charges rise with voltage for both settings; before 2 V, the $V_{d1}$ = $V_{d}$ setting has a higher peak transient current and total number of collected charges, but this is not significant; after 2 V, the situation is the opposite, and the two curves set by $V_{d1}$ = $V_{d}$ are significantly lower than the two curves set by $V_{d1}$ = 0 V. This shows that, for different operating voltages, they can be set differently. For the drain voltage greater than 2 V, $V_{d}$ = $V_{d}$ can be set, and, for the case of less than 2 V, the difference between the two cases is not significant; you can set $V_{d1}$ = 0 V, but taking into account the requirements of proportional reduction and low power consumption in advanced technology, $V_{d}$ is generally less than 2 V, and $V_{d1}$ = 0 V can be set at this time.

## 4. Summary

This paper proposes device design ideas for electric field mitigation in the sensitive region and charge collection shunt based on the single-particle effect charge collection process. To achieve this, a lightly doped source–drain end-system pn junction composite MOS structure is proposed. The device structure mitigates the phenomenon of driving current decrease with the growth in the relatively LDD region and further improves the SEE resistance of the device.

The structure’s device modelling was performed using TCAD Sentaurus 2018. The device’s electrical performance and single-particle resistance were simulated. The simulation results demonstrate that the relatively LDD_pn structure significantly enhances the driving current of the device compared to the normal and long_relatively LDD reference devices while also improving the device’s irradiation resistance under heavy-ion incidence conditions. Additionally, the number of charges collected at the leaky end is reduced by 25.23% and 13.85%, respectively. These findings suggest that the relatively LDD_pn structure has a good capability to resist single-particle devices.

The simulation modelled the single-particle effect under various conditions, including different LET values, positions, incidence angles, and sizes and doping concentrations of P-type regions. By comparing the number of charges collected by the devices under different conditions, the strong ability of the structure in this paper to resist single-particle effects is fully illustrated, and the feasibility of the principle of electric field mitigation and the idea of anti-irradiation reinforcement by shunt mechanism is verified.

## Figures and Tables

**Table 1:** 28 nm relatively LDD_pn structure NMOS device parameters.

Parameters	Value
Gate length/width	28 nm/1 μm
Substrate doping concentration (P-type)	1 × 10^15^ cm^−3^
Channel doping concentration (P-type)	3 × 10^18^ cm^−3^
Source–drain doping concentration (N-type)	4 × 10^20^ cm^−3^
RELATIVELY LDD doping concentration (N-type)	1 × 10^19^ cm^−3^
Secondary RELATIVELY LDD doping concentration (N-type)	5 × 10^19^ cm^−3^
RELATIVELY LDD length	36 nm
Thickness of the gate oxide (SiO-2/HfO-2)	2 nm/2.2 nm
P-region doping concentration on RELATIVELY LDD	3 × 10^18^ cm^−3^
Size of P area on RELATIVELY LDD (h/x)	10 nm/10 nm

**Table 2:** Default parameters of heavy-ion model and other physical models for simulation.

Parameters of the Heavy-Ion Model	Value	Other Physical Models
Direction	(0, −1, 0)	Fermi
Location/μm	(0, 0, 0)	Hydrodynamic (eTemperature)
Time/s	2 × 10^−11^	eQuantumPotential
Length/μm	0.05	SRH (DopingDependence)
Wt_hi/μm	0.015 μm	Auger
LET/(pC/μm)	1	Phumob/Enormal
Gaussian	PicoCoulomb	Enormal (Lombardi)

## Data Availability

The authors confrm that the data supporting the fndings of this study are available within the article.

## Abbreviations

The following abbreviations are used in this manuscript:

SEE	Single-Event Effects
NMOS	N-Metal-Oxide Semiconductor
MOS	Metal-Oxide Semiconductor
LET	Liner Energy Transfer
SRAM	Static Random-Access Memory
SOI	Silicon On Insulator
GAA	Gate All Ground
FinFET	Finned Field Effect Transistor
LDD	Lightly Doped Drain
TCAD	Technology Computer Aided Design
FWHW	Full Width at Half Maxima
